# Supplementary material for: Microbial coexistence through chemical-mediated interactions
Source: Nat Commun. 2019 May 3;10:2052. doi: 10.1038/s41467-019-10062-x (PMC6499789; doi:10.1038/s41467-019-10062-x)
Supplement: Supplementary file 1 — Supplementary Information [file 41467_2019_10062_MOESM1_ESM.pdf]

Supplementary Information

Microbial coexistence through chemical-mediated interactions

Niehaus et al.

Supplementary Figures and Tables

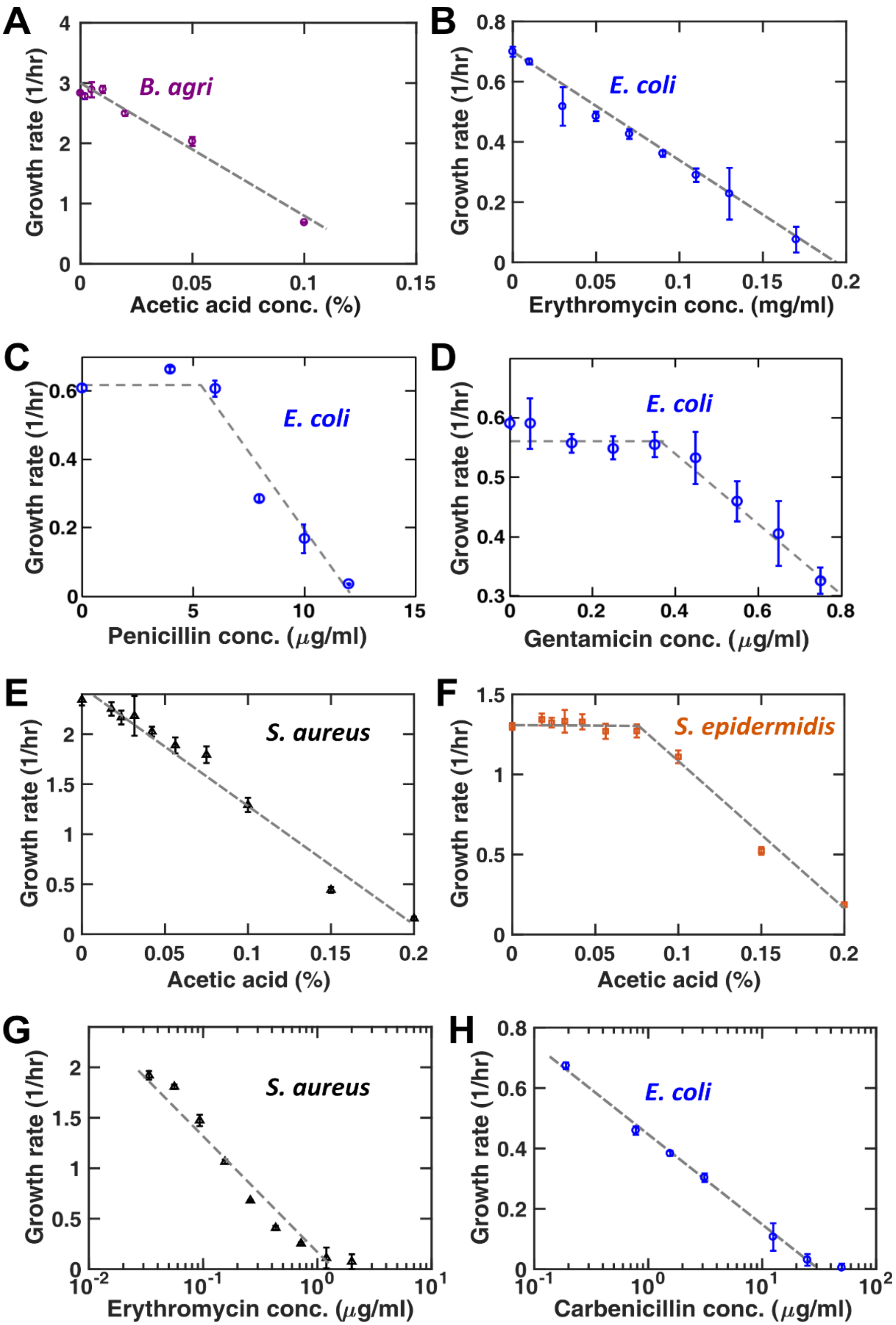

Supplementary Fig 1. Inhibition by chemical compounds often shows a consistent pattern of linear decrease, beyond a threshold concentration. Examining the growth inhibition by chemical compounds in many cases we have examined showed a linear decrease as the inhibitor concentration increased. (A) and (B) show two examples of this trend for *B. agri* and *E. coli*, in response to acetic acid and erythromycin, respectively. In these cases we observed a small threshold effect (i.e. little or no inhibition at very small concentrations of the inhibitor), and a linear decrease in growth rate, i.e. $\boldsymbol{dS/dt = (r_0 - \eta C)}$ offered a good approximation. (C) and (D) In some cases, we observed a more pronounced threshold effect, for example when *E. coli* was exposed to penicillin or gentamicin. This means these bacteria can tolerate the inhibitor to some extent, but beyond the threshold concentration, C_{th}, cells will be affected by the chemical inhibitor. For these situations, a more accurate model is $\boldsymbol{\frac{dS}{dt} = \begin{cases} r_0 S & ; C \leq C_{th} \\ r_0 S - \eta S(C - C_{th}) & ; C > C_{th} \end{cases}}$. (E) and (F) Other species and strains showed the same overall trends in many cases. As representative examples, growth rates of *S. aureus* and *S. epidermidis* in response to acetic acid show linear decrease (similar to (A-B)) and linear decrease beyond a threshold (similar to (C-D)), respectively. (G) and (H) Although the trends in (A-F) were most common, a third pattern was observed at lower frequency in which the decrease in growth rate was proportional to the logarithm of the inhibitor concentration. For simplicity, we chose the more common linear decrease trend in our simulations. Error bars show s.d. among 3-6 replicates.

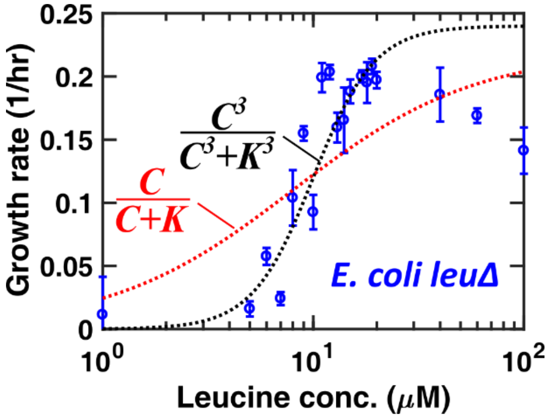

Supplementary Fig 2. Facilitation by chemical compounds shows a consistent pattern of saturable response. Similar to Fig 1D, we have observed that the influence of a facilitative chemical compound on species can be approximated by a Moser growth model. In this case, a leucine auxotrophic strain of *E. coli* is grown in defined M9 media supplemented with different concentrations of leucine. To avoid potential effects of leucine depletion, growth in the first 3 hours of growth is used to estimate the growth rate. Experimental observations of this auxotrophic strain suggest that a third-order relation (black dotted line) offers a more accurate estimation compared to the first-order Monod-type equation (red dotted curve). For simplicity, the first-order Monod-type equation is used in the model which still captures the saturating form of the equation. Similar to Fig 1D, at much higher concentrations of leucine, potential toxic effects reduce the growth rate (above 50 µM of leucine), but we do not include this effect in our model. Error bars show s.d. among 6 replicates.

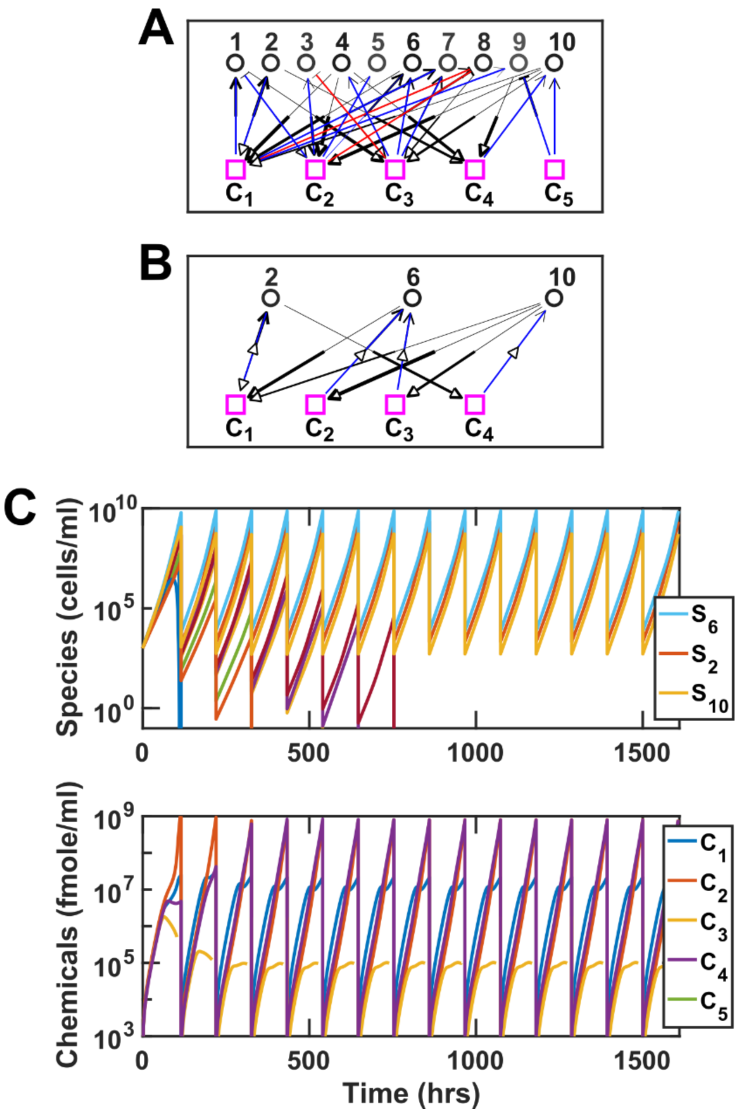

Supplementary Fig 3. During enrichment a subset of species can achieve coexistence. We show an example of the dynamics and network of a community assembled through enrichment. In (A) the random network of interactions in the initial pool of 10 species and 5 mediators is shown. (B) After enrichment, 3 species and 4 mediators remain in the community. In (A) and (B), the thickness of f-links indicates their relative strength and the thickness of c-links indicates the relative rate of removal or production. The shades of species indicates their relative basal growth rate (darker shades for higher basal growth rate values). Additionally, for f-links, those removed by their recipients are marked as blue (depletable) and those not removed by their recipients are marked as red (reusable). In this example, the initial ratio of positive to negative influences is 80%:20%, and the ratio of depletable to reusable mediator links is 80%:20%. (C) Dynamics of species population sizes (top) and chemical concentrations (bottom) are shown for the example of transition from (A) to (B). All populations are assumed to have a similar size at the beginning of enrichment. Any population that drops below 0.1 cells/ml is assumed to be extinct and is removed from the rest of the simulation. In this example, 3 species achieve coexistence, and coexistence appears to be stable after 7 rounds of growth-dilution. Other simulation parameters are similar to Fig 2 (listed in Methods).

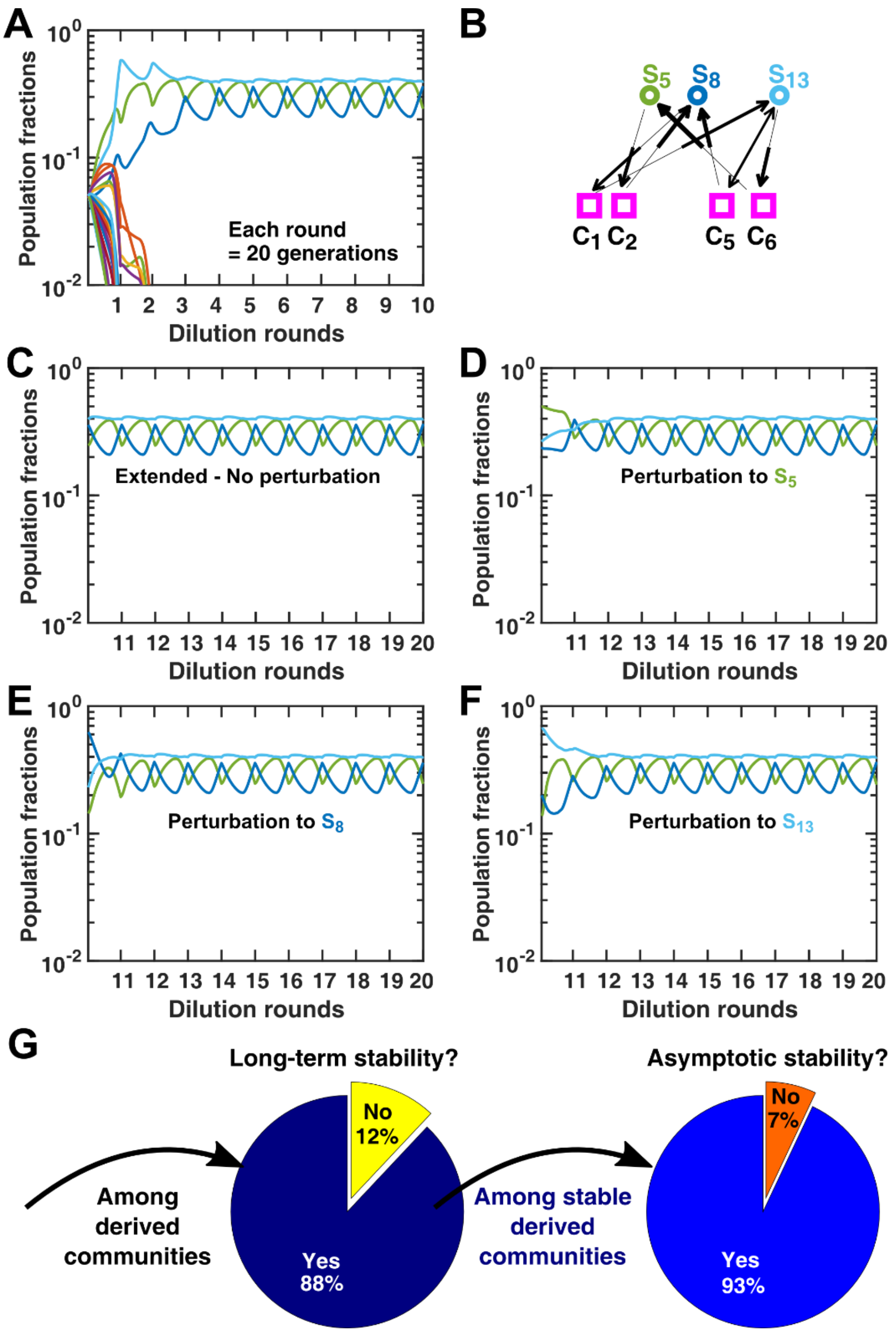

Supplementary Fig 4. Coexistence is closely related to "long-term stability" and "asymptotic stability". We examined long-term stability by extending the range of growth without introducing any perturbations. Here each round corresponds to 20 generations of community growth. In a representative example (A), enrichment leads to a derived community that shows coexistence of three species (B). In most cases (12% ± 7% s.d. among 1650 cases examined) communities that showed coexistence also exhibited long-term stability (G, left), similar to the example in (C). We also examined the asymptotic stability of community compositions, by changing the fraction of one of the member species by 3x and asking whether the community composition would recover to the initial fractions

afterwards (D-F). Recovery was assessed based on whether after 200 additional generations of community growth, the difference between fractions of species in the unperturbed versus perturbed community was less than 10%. Among communities that exhibit long-term stability, a majority of communities also demonstrated asymptotic stability (93% ± 5% s.d. among 1446 cases examined).

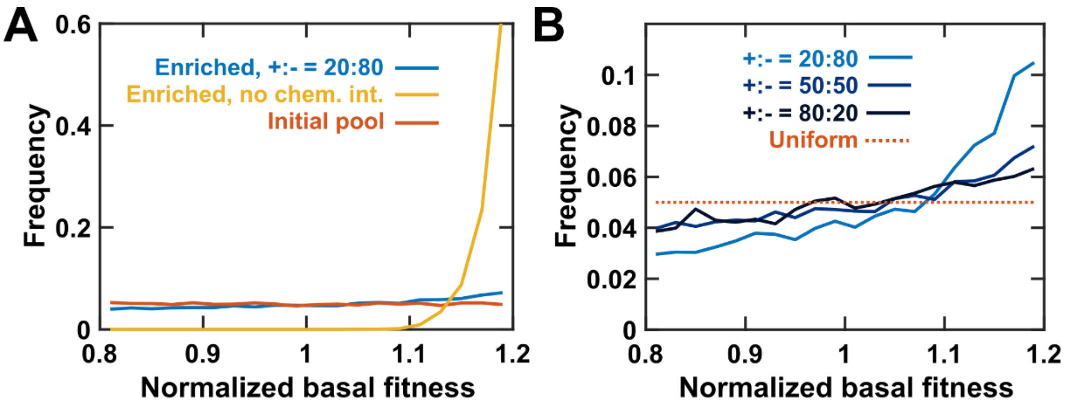

Supplementary Fig 5. Basal growth rate is a major determinant of coexistence in the absence of interactions, but not when species are engaged in strong interactions. (A) Examining the distribution of basal growth rate in all derived communities, we observe a slight advantage for species with higher basal growth rate in communities obtained from a pool of interacting species. In the absence of interactions (representing neutral theory), species with the highest basal growth rate will outcompete other species. The distribution in the initial pool is uniform across all basal growth rate values. These distributions are plotted as histograms of frequencies over 20 bins. (B) Comparing the distribution of basal growth rate in derived communities, we observe that although higher basal growth rates increase the chance of being present in derived communities, this trend is more pronounced if initial pools were dominated by inhibitory influences compared to facilitative influences. The dotted line shows the theoretical distribution of basal growth rate values in the initial pool. These distributions are plotted as histograms of frequencies over 20 bins. In simulations in (A) and (B), the average influence strength relative to average basal growth rate, $\boldsymbol{\rho/\sigma_{r0} = 20}$. The number of coexistence cases examined is 5000.

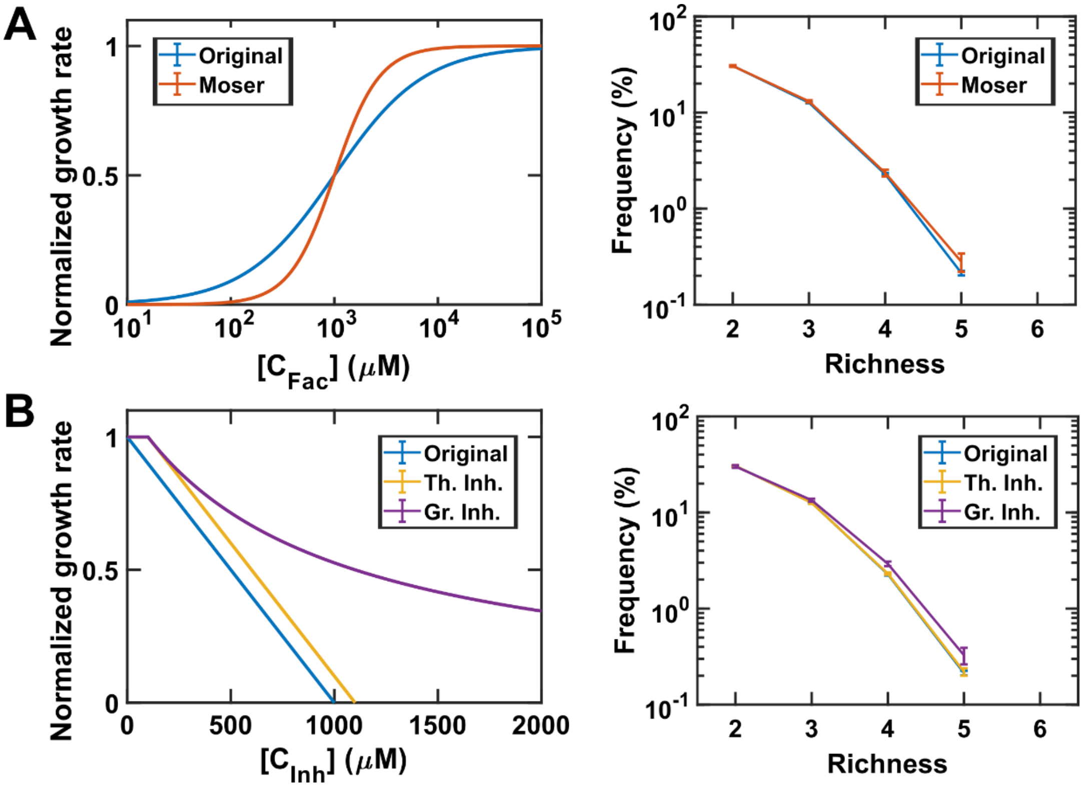

Supplementary Fig 6. Coexistence outcome is not sensitive to the detailed formulation of how chemicals affect the growth rate of species. We compare different assumptions of how mediators affect the growth rate of species. (A) Whether facilitation depends on the mediator as $\boldsymbol{C_l/(C_l + K_{i,l})}$ or $\boldsymbol{C_l^2/(C_l^2 + K_{i,l}^2)}$ (marked as Original versus Moser, respectively; left panel), does not significantly change the likelihood of coexistence (right panel). (B) We compared three ways of formulating inhibition by mediators (left panel): linear drop (Original; e.g. Supplementary Fig 1A-B), linear drop beyond a threshold (Th. Inh.; e.g. Supplementary Fig 1C-D), and the formulation in [1] that incorporates both the transfer of inhibitors inside the cell and lower sensitivity to inhibitors at smaller growth rates (Gr. Inh.). Likelihood of coexistence was not significantly different between 'Original' and 'Th. Inh.' cases. 'Gr. Inh.' showed a slightly increased likelihood of coexistence, presumably because on average the effect of inhibition in this case is lower. In these simulations, the average interaction strength relative to average basal growth rate, $\boldsymbol{\rho/\sigma_{r0} = 20}$, the number of initial species types N_c=10, the number of mediators N_m=5, and the number of initial pools examined N_s=5000. Here, the initial pool has a binomial network and contains similar fraction of positive and negative influences (+:- = 50%:50%).

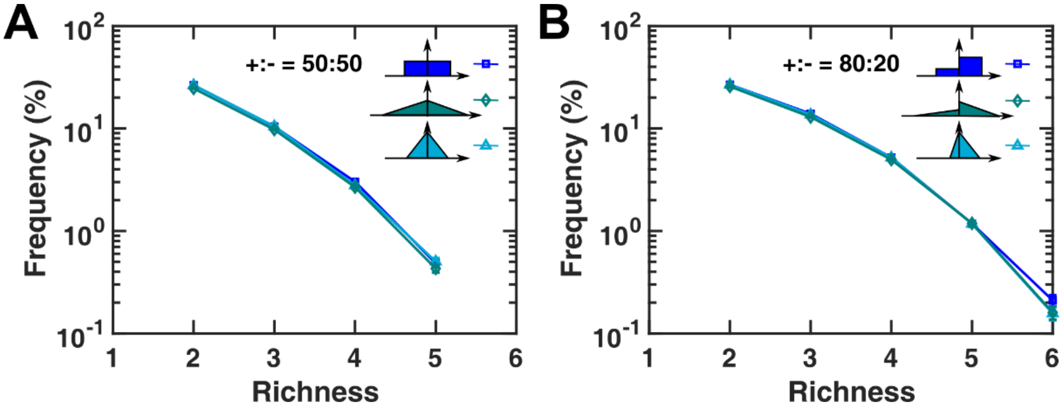

Supplementary Fig 7. Coexistence outcome is not sensitive to the distribution of influence strength values. Examining how the distribution of influence strengths in the initial pool affects coexistence, we find that the likelihood of coexistence is only modestly affected by the distribution of influence strengths. We examined situations where strong and weak interactions were equally likely (blue squares), where strong interactions were less likely (green diamonds), as well as a situation in which the less likely interaction type (positive or negative) has lower maximum influence strength (cyan triangles). Comparing the frequency of arriving at communities with different richness through enrichment shows a negligible difference in the outcome when different influence strength distributions are used when assigning interactions in the initial pool. Only simulation results with depletable mediators are shown, when (A) positive and negative influences are equally likely, and (B) positive influences are more likely than negative influences. In these simulations, the average influence strength relative to average basal growth rate, $\boldsymbol{\rho/\sigma_{r0} = 10}$. Error bars indicate s.d. due to sampling.

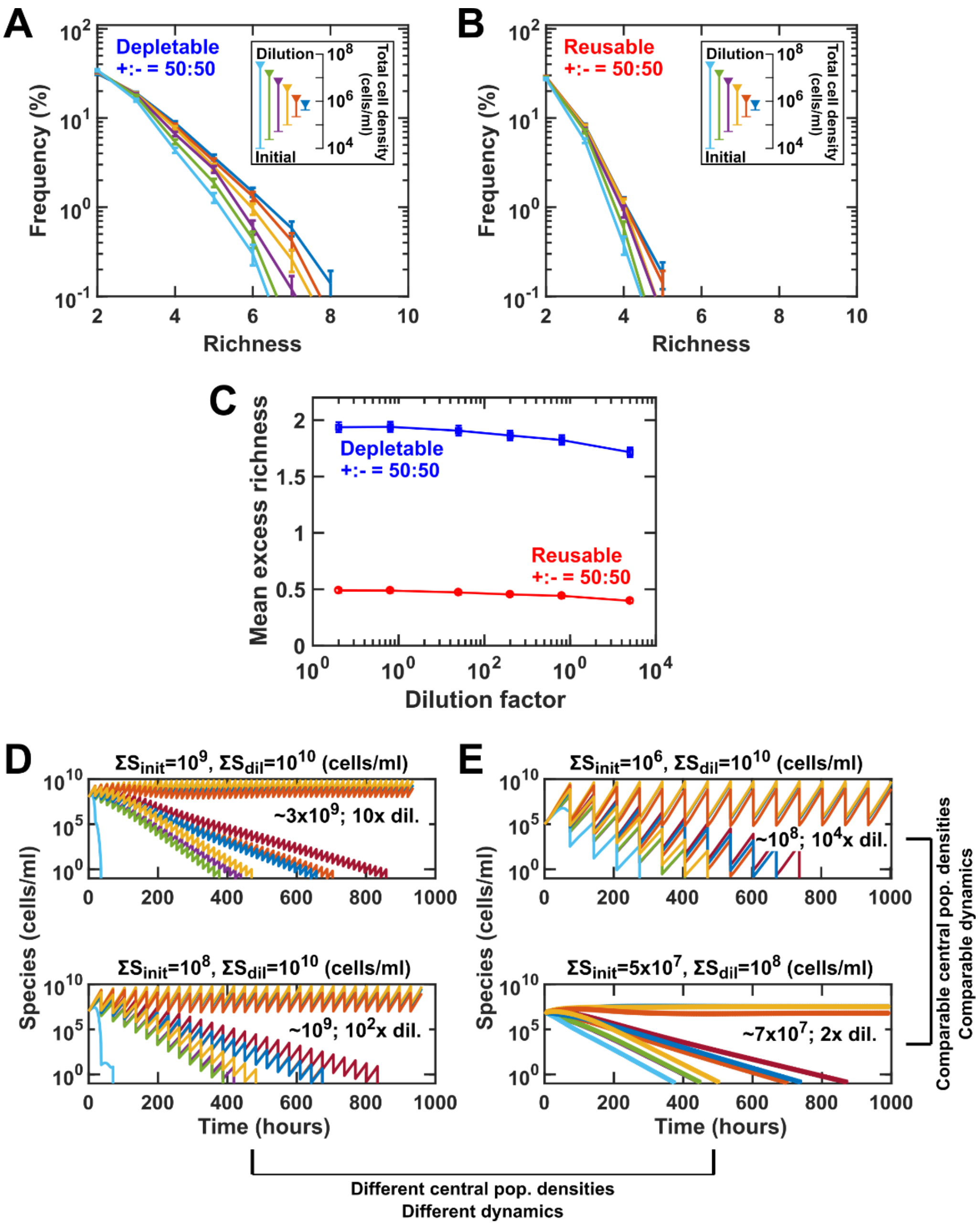

Supplementary Fig 8. Coexistence is not sensitive to the dilution scheme. The likelihood of coexistence is only modestly affected by the dilution factor (i.e. the ratio of total population density before and after dilution). (A-B) As the range of population growth around a central population density expands (insets) from a 2-fold range (from 5×10^5 to 10^6 cells/ml) to a 5000-fold range (from 10^4 to 5×10^7 cells/ml), coexistence decreases, but gradually. (C) Mean excess richness measure (defined as average richness in derived communities, beyond competitive exclusion) confirms a gradual drop in coexistence when the dilution factor increases. Error bars show bootstrap estimates of 95% confidence intervals for the mean values. In these simulations, $\boldsymbol{\rho/\sigma_{r0} = 20}$, the number of initial species types N_c=20, the number of mediators N_m=10, and the number of initial pools examined N_s=5000. Using a representative example with N_c=10 and N_m=5, we observe that (D) increasing the dilution step from 10x (top) to 100x (bottom) does not considerably change the population dynamics in the community, when the central population density does not change much. Further increasing the dilution step to 10^4x (E, top) leads to some

qualitative changes, such as persistence of the cyan population beyond 200 hours. This is because the inhibition of the cyan population is weaker in (E), in which there is lower inhibitor concentration (and fewer inhibitor producers) at the beginning of each round. When we shift the densities to the center of the range in (E, top), dynamics become qualitatively similar (E, bottom), even with a gentle 2-fold dilution. Total central cell density (cells/ml) and fold-dilution are marked in the inset of each panel.

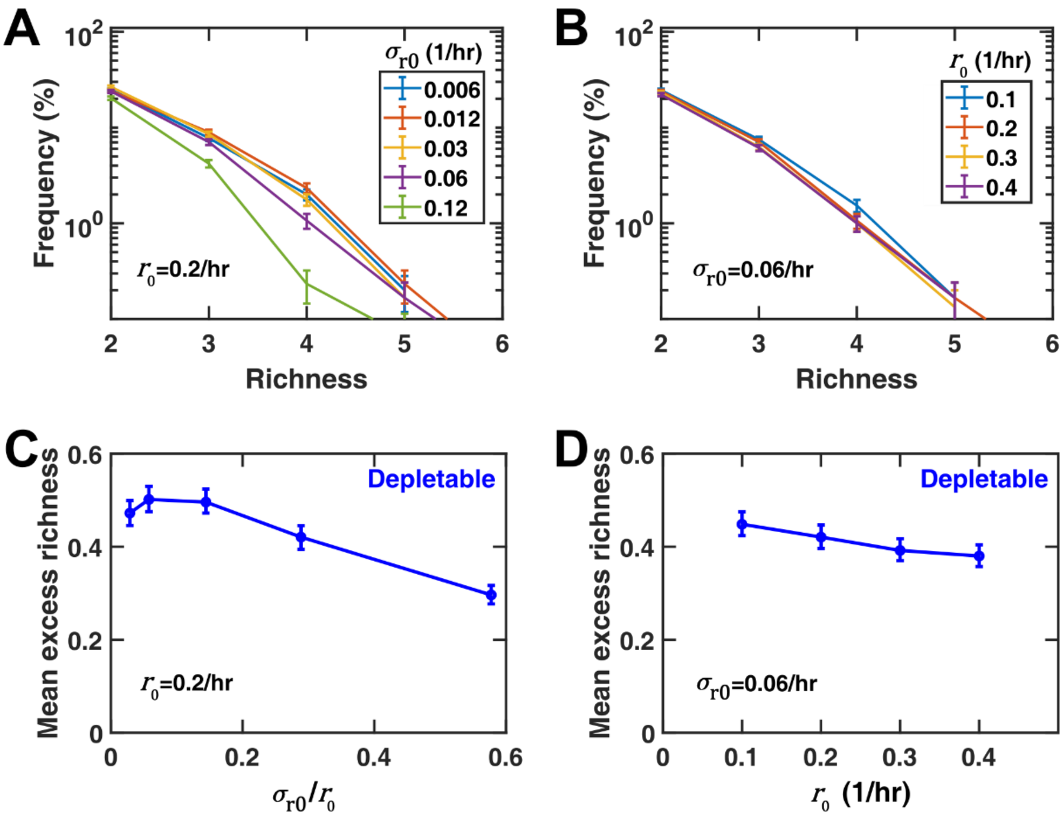

Supplementary Fig 9. Coexistence is sensitive to the standard deviation of the basal growth rates in the initial pool. (A) As the standard deviation of basal growth rates in the initial pool ($\boldsymbol{\sigma_{r0}}$) increases, the likelihood of coexistence decreases. The mean of basal growth rates ($\boldsymbol{r_0}$) is kept constant in these cases. (B) The likelihood of coexistence appears to be fairly insensitive to the mean basal growth rate in the initial pool, when $\boldsymbol{\sigma_{r0}}$ is kept fixed. Error bars in (A) and (B) are standard deviations based on counting uncertainty. (C-D) Mean excess richness values (average number of coexistent species beyond competitive exclusion) agree with qualitative trends in (A) and (B). Error bars in (C) and (D) show bootstrap estimates of 95% confidence intervals for the mean values.

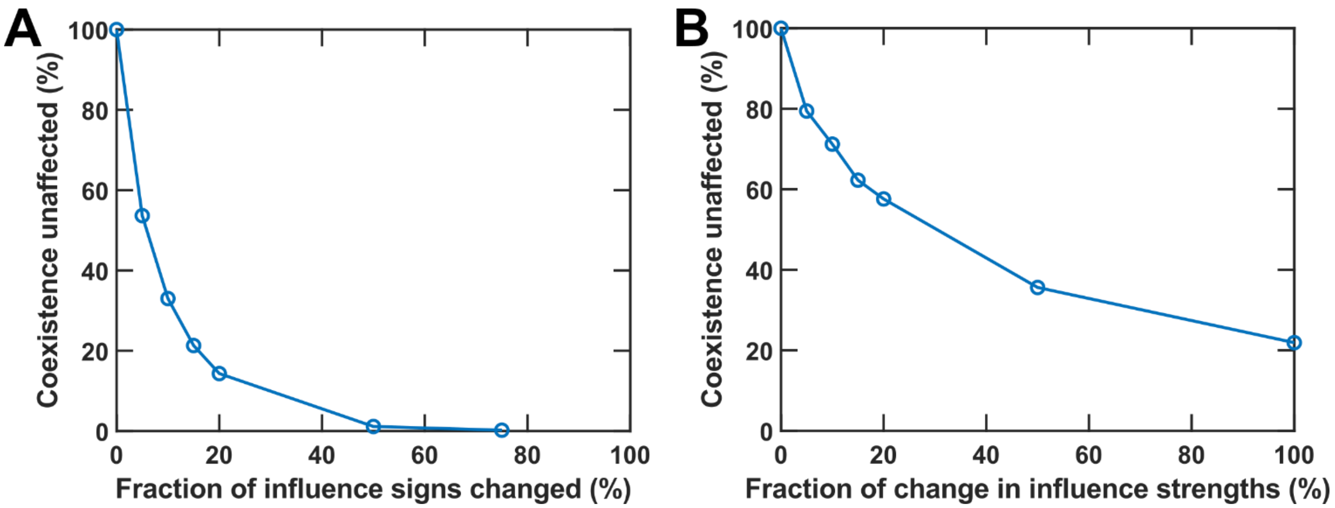

Supplementary Fig 10. Coexistence is more sensitive to the signs and less sensitive to the values of influence strengths. (A) Given a network topology and without changing the strengths of influences, we changed the signs of a fraction of influences in the initial pool. The results were quite sensitive, with changing only 5% of influences affecting coexistence in 46% of cases. (B) Given a network topology and without changing the signs of influences, we changed the strengths of all influences in the initial pool. In this plot, a 20% change in strength means all influence strengths are randomly either increased by 20% or decreased by 20%) The results were less sensitive (compared to a change in sign). Simulation parameters are similar to Fig 2. Here +:- = 50%:50% is assumed.

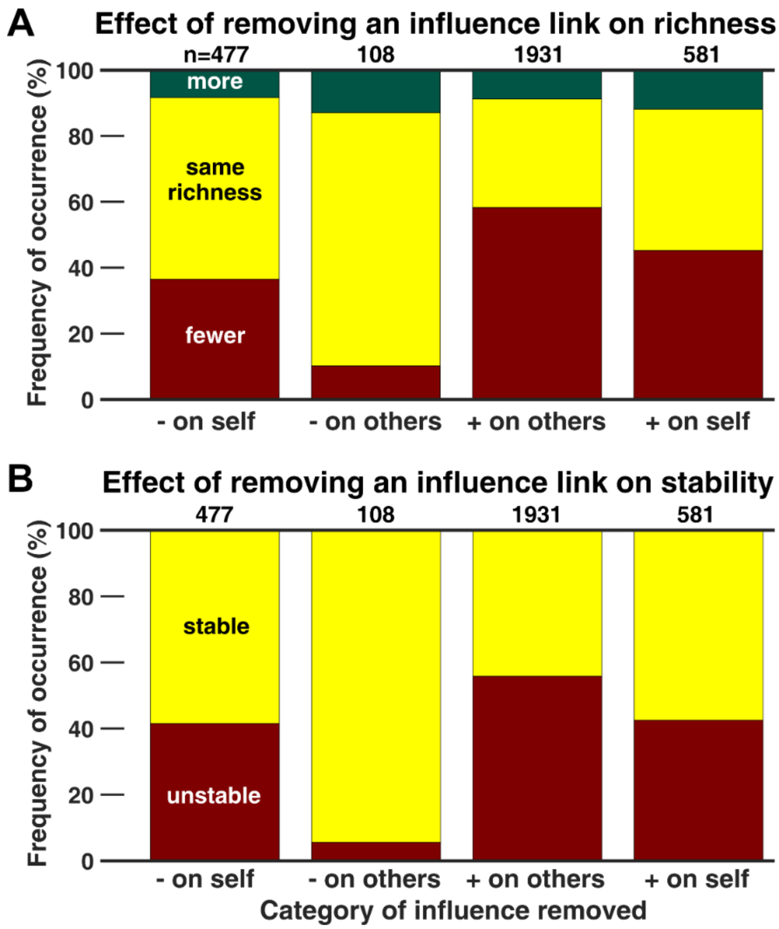

Supplementary Fig 11. Individual influences can impact coexistence and stability regardless of the interaction makeup in the initial pool. We use knock-out experiments, similar to Fig 5 to assess how removing a link from the interaction network affects coexistence and stability. Here, the initial pool has a binomial network and contains mostly negative influences (+:- = 20%:80%). The conclusions are similar to Fig 5. (A) Removing facilitation links likely disrupts coexistence whereas removing inhibition of others is unlikely to break down coexistence. (B) Removing facilitation likely disrupts the stable community, whereas removal of inhibition of others is unlikely to impact the community.

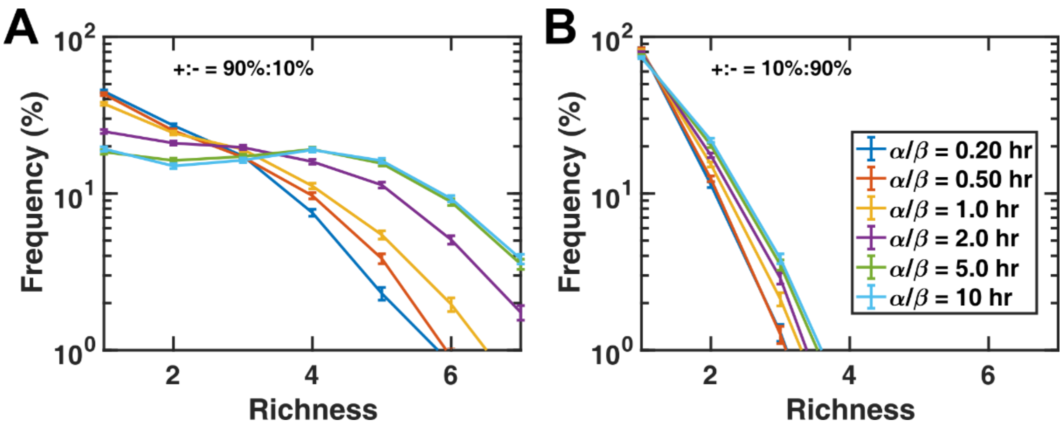

Supplementary Fig 12. Depletion of mediators favors coexistence. We examined how changes in average consumption and production rates of chemical mediators (α and β, respectively) affected coexistence. For both communities that contained many positive influences (A) and those that contained many negative influences (B), an increase in the ratio of α/β increased the likelihood of coexistence. This trend was more pronounced in communities that had more positive influences (A), but was saturated beyond a ratio of 5. In these simulations, the initial number of species types N_c=20, the number of possible mediators N_m=15, and a binomial network connectivity is assumed with q_p=0.2 and q_c=0.2. The number of communities analyzed N_s=5000. Error bars indicate s.d. due to sampling.

Supplementary Table 1. Matlab scripts used for modeling and analysis of data are listed.

Code	Description
`Enrichment_WM_DpMM.m`	Simulates the enrichment process for communities with given parameters using the model that explicitly includes chemical mediators. As output, it creates an ensemble of communities that show coexistence For the same network connectivity, different ratios of +:- influences and different fraction of depletable versus reusable mediators are simulated for side-by-side comparison.
`DistInteractionStrengthMT_PB.m`	Produces a matrix of influence strengths with uniform distribution of strengths and designated probability of positive versus negative signs.
`Dynamics_WM_DpMM.m`	Simulates the dynamics of a well-mixed community of species interacting via chemical mediators
`NetworkConfig_Binomial.m`	Produces matrix of links for a network with binomial connectivity

References

1. Scott M, Gunderson CW, Mateescu EM, Zhang Z, Hwa T. Interdependence of cell growth and gene expression: origins and consequences. Science. American Association for the Advancement of Science; 2010;330: 1099–102. doi:10.1126/science.1192588
